# Context-aware monitoring: rethinking comprehensive screening in the era of AI

**DOI:** 10.1038/s41746-026-03106-2

**Published:** 2026-08-13

**Authors:** David A. Shaywitz, Nathan D. Price, Daniel K. Sodickson

**Affiliations:** 1 Department of Biomedical Informatics, Harvard Medical School, Boston, MA USA; 2 Buck Institute for Research on Aging, Novato, CA USA; 3 Thorne HealthTech, New York, NY USA; 4 Center for Advanced Imaging Innovation and Research (CAI2R), Department of Radiology, New York University Grossman School of Medicine, New York, NY USA; 5 Function Health, Austin, TX USA

**Keywords:** Computational biology and bioinformatics, Diseases, Health care, Mathematics and computing, Medical research

## Abstract

Applying advanced measurement technologies proactively in asymptomatic populations predictably yields false positives, as a consequence of Bayes’ Theorem. Yet the same Bayesian arithmetic suggests a remedy: adding context. Serial and multimodal measurements, integrated using context-aware AI that prioritizes within-person change over population norms, can reduce false positive rates while preserving sensitivity. We describe early illustrations from imaging and molecular diagnostics and discuss challenges including cost, anxiety, liability, and equity.

Most diseases of aging, including cardiovascular illness, neurodegeneration, and many types of cancers, develop gradually over years, offering an attractive window for early detection and potential mitigation. The explosive development of ever-more sophisticated measurement technologies, from imaging to -omics to wearables, has provided the opportunity to collect dense molecular, structural, and physiological data, while rapid advances in AI offer powerful analytic capabilities to help parse these multimodal parameters.

The promise of advanced detection of a wellness-to-disease transition has attracted many adults to consumer health platforms that offer comprehensive testing^1^. This trend worries many doctors, who learned in their medical school biostatistics coursework that extensive testing of unselected individuals for uncommon conditions generally produces far more false alarms than actionable signal – a basic consequence of Bayes’ Theorem. Concerned physicians point to the challenge of “incidentalomas,” inadvertently discovered anomalies that (while typically benign) can increase patient anxiety, spawn elaborate workups, and introduce the possibility of iatrogenic harm^2^.

The result is a dilemma: how to productively leverage our increasingly rich collection of measurement tools and analytic technologies to detect early signs of disease without incurring the penalties that can seem inextricably bound up with such comprehensive testing?

## Potential advantages of adding context

Fortunately, the same math that underlies this tension may also offer a solution. Modeling indicates that while drawing conclusions from selected measures captured at a single time point is unquestionably fraught, prediction materially improves if an individual’s data are captured serially. Bayes’ Theorem suggests that the reliability of screening tests can be improved simply by repetition, so long as estimates of the prior probability of disease are updated appropriately ^3^. Under these assumptions, even just a few time points can reduce false-positive classifications and increase the effective specificity of a screening strategy; within-person change may provide a more informative signal than one-shot population norms, which incorporate a high degree of individual variability. Of note, information gained from serial measurements is attenuated when successive tests share common sources of noise or biological variability, violating the conditional independence assumption implicit in naïve Bayesian updating^4^. Real world gains in specificity will therefore be smaller than theoretical maxima, underscoring the importance of combining complementary—orthogonal—data sources rather than repeating correlated measurements. Decision-making accuracy can also be improved by evaluation of mechanistically linked parameters (such as diverse biomarkers) that are expected to move in a coordinated fashion if pathology is present, and by examining data from multiple modalities, such as imaging and molecular data. Such approaches are routinely utilized by clinicians in daily practice and in multidisciplinary clinics such as “tumor boards”.

In short: context – which can be provided by time, coordinated measures in the same domain, or data from multiple modalities—improves accuracy. However, physicians are currently limited both by the inconsistent availability of temporal and/or multimodal context and by the capacity of the human brain to assimilate large quantities of disparate contextual information. Consequently, systematic incorporation of individual context into automated medical assessments represents a powerful opportunity for patient care, and it is one that can be greatly expanded at scale with emerging AI tools. Several recent examples highlight how this might work.

## Early illustrations of the value of added context

As one of us has recently demonstrated, serial imaging of individuals, together with suitable AI, enables standard-of-care quality images to be obtained from radically less data, igniting a virtuous cycle: lower data requirements can reduce costs and enable imaging using less specialized equipment, in more accessible settings, facilitating the acquisition of individual data at still more time points^5^.

**Fig. 1:**
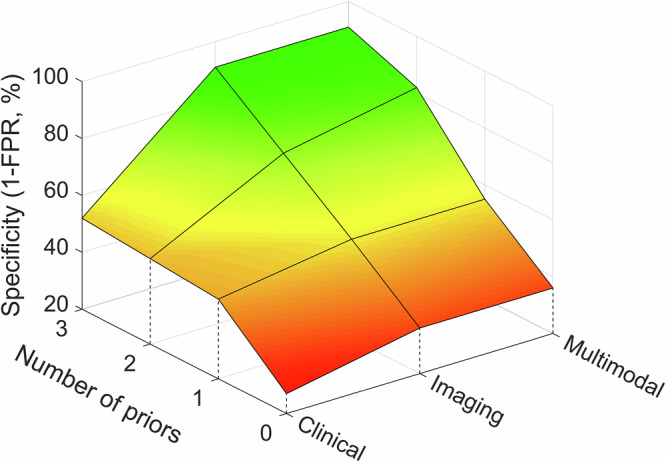
False positive rates are not fixed. The surface plot indicates specificity (one minus the false positive rate) for prediction of the risk of clinically significant prostate cancer five years following an index visit, using a deep learning model trained on data from a cohort of nearly 30,000 patients studied over the course of nearly a decade. The context-aware AI model incorporates varying types of prior data from varying numbers of previous visits. Specificity increases markedly as more priors and more varied priors are incorporated, growing from less than 30% to more than 90%, at a constant ~90% sensitivity. (Based on data from Umapathy L et al., arXiv: 2510.15591. Figure generated using MATLAB, MathWorks, Inc).

Recent work by the same group provides preliminary evidence consistent with this framework. In a retrospective analysis of a single-center cohort of nearly 30,000 patients followed for approximately a decade, AI models trained to predict the current and future risk of clinically significant prostate cancer showed progressive reductions in false positive rates at constant high sensitivity as additional context, in the form of prior imaging and/or clinical data, was provided to them^6^. Consistent improvements were seen both with clinical context and with imaging context individually, and still greater improvements resulted when both types of contextual data were incorporated together (see Fig. 1). These findings, if confirmed through external validation in diverse populations, would support the premise that multimodal longitudinal context can improve screening specificity. However, the results should be interpreted cautiously pending peer review and independent replication.

Note that the deep learning model described in Fig. 1 uses neural networks that learn complex temporal patterns from longitudinal data. While not explicitly Bayesian in architecture, this model achieves a functionally similar updating effect as additional context accumulates.

Combining the two uses of context described above—to enable more accessible testing and to improve the specificity of testing—could provide an approach to reduce the burden and increase the value of regular surveillance for diseases such as cancer.

A similar logic applies to molecular diagnostics. In prostate cancer, combining complementary markers can improve specificity at a given sensitivity. In a modeled analysis among men with PSA ≥3 ng/mL, using the Stockholm-3 model as a reflex test would have avoided 33% of biopsies while accepting a 10% reduction in detection of Gleason score ≥7 cancer, versus 14% avoided by simply raising the PSA threshold to the same sensitivity^7^. Parallel work with kallikrein (4K) panels shows similar value as a reflex test after elevated PSA. In a PSA-then-MRI screening pathway, adding 4Kscore before MRI would have reduced MRI use by 41%, biopsies by 28%, and low-grade cancer diagnoses by 23%, while delaying detection of 4% of grade group ≥2 cancers^8^. Both the S3M and 4K panels use conventional logistic regression; their innovation lies in which features to combine. Considered together, these results illustrate how joint evaluation of mechanistically related features constrains spurious elevations that would trigger interventions if each were viewed in isolation.

These approaches should be understood in the context of long-standing challenges in screening optimization. Prostate cancer screening has been shaped by decades of debate about overdiagnosis—the detection of indolent cancers that would never have caused symptoms or death. The ERSPC 23-year follow-up, while documenting a significant reduction in prostate cancer deaths among men invited to screening, also found 27 excess cases per 1000 men invited to screening, with a doubling of low-risk cancer detection in the screening group^9^; broader estimates suggest 20–50% of screen-detected cases represent overdiagnosis^10^. Earlier efforts to improve specificity through longitudinal PSA data—specifically PSA velocity and doubling time – were extensively studied and found to add little independent predictive value beyond PSA level alone^11^. The failure is instructive: PSA kinetics are mathematically derived from PSA itself, providing correlated rather than independent information. What distinguishes context-aware AI is the integration of mechanistically distinct data sources—imaging, molecular, and clinical—with nonlinear pattern recognition across within-person trajectories. Multi-cancer early detection tests such as Galleri (a cfDNA methylation-based assay^12^) represent a complementary approach to comprehensive screening, though the recent NHS-Galleri trial did not demonstrate a statistically significant reduction in late-stage cancer incidence at 3 years, underscoring the need to demonstrate clinical benefit in randomized trials^13^. Context-aware monitoring—through serial measurement and integration with orthogonal modalities—could address this gap. Empirical precedent from serial low-dose CT lung cancer screening illustrates how structured temporal context improves specificity, even without AI: false-positive rates declined from 26.3% at baseline to 15.9% by the third round in the NLST, and from 19.8% to 3.9% by year 5.5 in NELSON^14^.

Outside oncology, layering orthogonal biology and additional time points improves early detection and calibration. In pregnancy, cell-free RNA signatures that integrate placental and maternal signals identify risk of preeclampsia months before presentation and outperform models based on clinical risk factors; discovery-scale profiling was subsequently distilled to compact, implementable gene sets^15,16^. At population scale, UK Biobank proteomics shows how modern machine learning handles high dimensionality—learning which proteins add information, which are redundant, and which are noise^17^. This insight may then be used to identify parsimonious, easily-deployable signatures that improve 10-year disease prediction over clinical baselines.

It bears noting that the examples above span a range of evidence maturity— from validated reflex testing panels in clinical use (S3M, 4K) to early-stage research on longitudinal deep learning models—and each entails distinct regulatory, validation, and implementation requirements.

## Challenges and opportunities for practical implementation of context-aware monitoring

While the addition of context through repeated measurements and the consideration of a broader set of parameters may offer great value in the early and actionable detection of potentially pathological change, successful implementation will require careful attention to four prominent challenges.

### Cost

Routine adoption of regular context-aware monitoring will require testing that is less expensive and more convenient than many current diagnostic approaches. To some extent, costs can be reduced through economies of scale as well as through technological advances, as has been seen in the blood testing industry. In the arena of imaging, the possibility of using limited data from low-cost interval scans to detect changes from individual baselines dovetails nicely with a recent explosion in the development of accessible imaging technologies (such as portable low-field MRI systems, which have recently received FDA clearance). Moreover, the extra years of healthy life that context-aware monitoring could deliver may represent an intrinsically attractive value proposition for patients and society even in the absence of objectively lower costs. To be sure, the clinical benefit of context-aware monitoring must be established before cost-effectiveness can be established. Realizing the economic case will also require reimbursement frameworks— particularly shifts towards value-based care models—that incentivize prevention rather than treatment. Cost-effectiveness analyses of multi-marker approaches (e.g., Stockholm-3 with MRI^18^) suggest these thresholds are obtainable, but they have not yet been established for the broader context-aware monitoring paradigm we describe.

### Anxiety

A common objection to the idea of expanding surveillance is that it may generate anxiety for those who are inclined to worry about the problems that may come to light with each new test—though for others, particularly those who have experienced serious illness in people close to them, the same information may provide considerable reassurance. Much of the anxiety may relate to our current default paradigm of bringing diagnostic technology to bear at relatively late stages in disease, when symptoms are present, suspicions are high, and interventions may be too late to fix the problem. If the most common outcome of serial testing in the future is reassurance and successful care, then expectations may change. When the impact of genetic sequencing was evaluated in the BabySeq Project randomized clinical trial, the subjects did not experience a persistent increase in anxiety following the disclosure of newborn genomic sequencing results, including among the relatively small subgroup of parents whose newborn received a monogenic disease risk finding^19^. We note that the BabySeq finding involved a single disclosure event, and its applicability to repeated surveillance in asymptomatic adults has not been established. However, a meta-analysis of seven CSER Consortium studies found that anxiety and depression did not significantly increase following disclosure of exome/genome sequencing results^20^, and psychosocial evaluation within the PATHFINDER multi-cancer detection study found that while distress and uncertainty increased transiently following results disclosure, levels were low overall^21^. Nevertheless, as with any broadening of routine health monitoring, the psychological effects of iterative surveillance in healthy populations would benefit from dedicated longitudinal evaluation.

### Liability

Responsibility for the legal risk incurred through interval testing will need to be established. Clinicians are unlikely to pursue expansive evaluations for patients if in doing so, they are assuming an increased risk either of overdiagnosis or of missed diagnosis. Once again, AI may prove to be a valuable tool. While reliance on AI for current care-driving tasks raises a series of regulatory, ethical, and practical concerns, the use of context-aware AI for interval monitoring, whose function is principally to raise early warning flags with high specificity, may have a different balance of risks and rewards. In particular, because such tools flag cases for clinician review rather than dictating management, the interpretability requirements may be less stringent than for AI systems that directly guide treatment – though transparent reporting of what contextual features drive a given alert will remain important for clinical trust. Applied as proposed, AI could facilitate the wide deployment of established approaches to disease prevention in an earlier and more consistent fashion. Meanwhile, the regulatory landscape for interval AI monitoring remains undefined. The FDA has articulated a total product lifecycle approach for AI-enabled medical devices, including predetermined change control plans for adaptive algorithms that learn over time^22^. However, the appropriate classification for context-aware monitoring tools – aimed at providing early warning or suggesting ways to improve health and increase resilience – and the allocation of liability between AI developers, platform operators, and clinicians for signals that prove incorrect in either direction, represent significant unresolved challenges.

### Value, equity, and algorithmic bias

While the potential benefits of context-aware monitoring are compelling, physicians and payors alike will of course be looking to long-term studies to document and quantify those benefits. Consumer health platforms offer an important opportunity to assemble large-scale, longitudinal datasets of health trajectories. However, these data are subject to selection bias – often enriched for early adopters and more affluent users, whose trajectories may not be representative of the broader population. Thus, questions of equity and algorithmic bias warrant careful attention. AI systems trained on non-representative data can systematically underdiagnose underserved populations, as demonstrated in analyses of chest radiograph algorithms^23^. The studies cited in our discussion derive predominantly from academic centers in high-income countries and performance in underrepresented groups remains largely untested. The evidence ultimately needs to include prospective studies demonstrating that context-aware monitoring leads to earlier intervention, reduced morbidity, and net clinical benefit across diverse populations. Context-aware AI could in principle reduce bias by using individual baselines rather than population norms, but this benefit will only be realized if the underlying data and algorithms are developed with diverse training sets and equity-focused evaluation.

Ultimately, the case for context-aware monitoring rests on a principle whose theoretical foundation is strong, but whose clinical validation remains incomplete. The same Bayesian mathematics that generates the false positive problem in comprehensive screening also points towards its resolution through serial, multimodal assessment—but translating this from theory to practice will require rigorous, inclusive evaluation that has not yet been conducted. As the ability to collect denser and richer longitudinal data continues to expand, and AI analytics continue to improve, there is a growing incentive to invest in the prospective studies, regulatory frameworks, and equity-focused implementation needed to determine whether and how these technologies can be harnessed effectively. If the evidence confirms the promise suggested by early results, context-aware monitoring could meaningfully improve our ability to detect encroaching disease while there is still time for productive intervention—a possibility that merits sustained investigation.

## Data Availability

No datasets were generated or analysed during the current study.
